# The protective effects of alpha-pinene on high glucose-induced oxidative stress and inflammation in HepG2 cells

**DOI:** 10.22038/IJBMS.2024.74546.16191

**Published:** 2024

**Authors:** Razieh Choghakhori, Mojgan Azadpour, Amir Abbasnezhad, Farzad Ebrahimzadeh, Hassan Ahmadvand

**Affiliations:** 1 Razi Herbal Medicines Research Center, Faculty of Medicine, Lorestan University of Medical Sciences, Khorramabad, Iran; 2 Nutritional Health Research Center, Lorestan University of Medical Sciences, Khorramabad, Iran; 3 Medicinal Plants and Natural Products Research Center, Hamadan University of Medical Sciences, Hamadan, Iran

**Keywords:** Alpha-pinene, Cell viability, Hyperglycemia, Inflammation, Oxidative stress

## Abstract

**Objective(s):**

Hyperglycemia, a prevalent metabolic condition observed in diabetes, leads to oxidative damage, inflammatory responses, and other consequences. Natural compounds alleviate the adverse impacts of diabetes. We aimed to explore the effects of alpha-pinene (AP) as a monoterpene on oxidative damage and inflammation caused by high glucose (HG) in the human hepatocellular liver carcinoma (HepG2) cell line.

**Materials and Methods:**

The HepG2 cells were subjected to non or HG concentration (50 mM) and treated with or without AP (8, 16, and 32 μg/ml) for 48 hr. The effect of treatments on cellular viability, malondialdehyde (MDA), glutathione (GSH), and activity of anti-oxidant enzymes, including glutathione peroxidase (GPx), catalase (CAT), superoxide dismutase (SOD), was determined. The gene expression levels of nuclear factor-κβ (NF-κB), tumor necrosis factor-alpha (TNF-α), interleukin-6 (IL-6), and dipeptidyl peptidase-4 (DPP-4) were estimated using quantitative real-time polymerase chain reaction (qRT-PCR).

**Results:**

HG exposure significantly increased cell death, MDA formation, and depletion of GSH content and GPx, CAT, and SOD activity (*P*<0.05). We have also seen a significant induction in NF-κB, TNF-α, IL-6, and DPP-4 gene expression in hepatocytes under HG conditions (*P*<0.05). Interestingly, co-treatment with AP in a dose-dependent manner improved cell death and altered levels of MDA and GSH, and activity of GPx and CAT (*P*<0.05). AP could also modulate the gene expression of NF-κB and inflammatory biomarkers dose-dependently (*P*<0.05).

**Conclusion:**

Our findings suggested the protective effect of AP on hepatocytes under HG conditions through attenuating oxidative stress markers and suppression of inflammatory pathways.

## Introduction

Chronic hyperglycemia has a critical role in the occurrence of metabolic disorders, including type 2 diabetes (DM), metabolic syndrome (MS), and obesity, which can lead to a variety of complications such as micro- and macro-vascular dysfunction and multi-organ failure (1, 2). High glucose levels cause cellular failure due to increased reactive oxygen species and decreased anti-oxidant content (3). Lipid peroxidation, a hallmark of oxidative stress, occurs from ROS attaching to unsaturated fatty acids, altering their structure and producing malondialdehyde (MDA) (4). Under physiological situations, numerous mitochondrial, cytosolic, and peroxisomal anti-oxidant systems, including superoxide dismutase (SOD), catalase (CAT), glutathione peroxidase (GPx), and tissue glutathione (GSH) content, are implicated in reducing the destructive potential of oxidative stress in cells (5, 6). Thus, a balance might be important between ROS and the levels of anti-oxidants. It is widely evidenced that chronic hyperglycemic induced low-grade inflammation subsequently plays a key role in the onset and progression of metabolic diseases. The transcription factor NF-B pathways have been implicated in developing oxidative stress-mediated inflammatory responses in hyperglycemia (7). The transcription factor NF-κB promotes immunity through induction of pro-inflammatory genes involved in inflammation, such as tumor necrosis factor-alpha (TNF-α) and interleukin-6 (IL-6) (7, 8). Moreover, dipeptidyl peptidase-4 (DPP-4) is a cell surface serine protease that exhibits widespread tissue distribution and is highly expressed in the liver in DM (9). The previous findings demonstrated that HG levels have been consistently associated with enhanced DPP-4 expression and activity (9, 10). Increased liver DPP-4 activity leads to chronic liver inflammation through induction of oxidative stress and activation of the NF-κB signaling pathway (11). So, blocking these mediators could be a practical approach to regulating hyperglycemia-induced oxidative stress and inflammation. 

**Figure 1:**
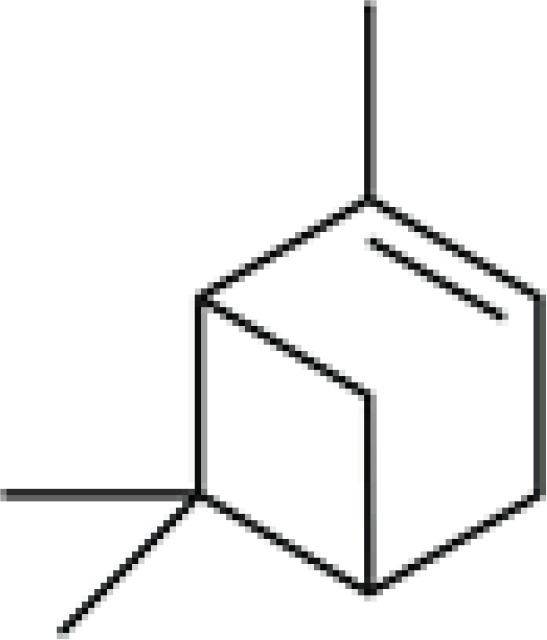
Chemical structure of the alpha pinene

Natural products, as effective and cost-efficient therapeutic molecules, are the most important medicinal discovery, which is safe and well-tolerated by the cell system (12). They comprise biologically verified structural characteristics with pharmacological activities (12, 13). Terpenes have drawn the attention of pharmaceutical researchers as potent pharmacological ingredients due to their varying chemical configurations and biological features (14, 15). Alpha-pinene (AP) is a bicyclic hydrocarbon constituting of two units of isoprene with an overall formula C10H16 (Figure 1) (16). It is found in the essential oils derived from many species of herbs such as *genus Pinus*,* Pistacia atlantica*, *Eucalyptus*, *Rosmarinus officinalis*, *Camphor*, *Psidium*, *Bupleurum fruiticescens*, *Piper nigrum,* and *Juniperus*. AP has numerous pharmacological effects, including antifungal, antibacterial, insecticidal, anti-leishmanial, antiapoptotic, anti-inflammatory, anti-oxidative, and neuro- and gastro-protective activities (17, 18). 

Investigating the consequences of hyperglycemia in cultured liver cells would be interesting. Findings have shown that the human hepatoma cell line (HepG2) has been widely used to research hyperglycemia in vitro (7, 19-23). To the best of our knowledge, no studies have been conducted on the influences of AP on HG-induced oxidative damage and inflammation in an in vitro model. In the current study, we aimed to evaluate the protective impacts of AP on HG-induced oxidative damage and inflammatory response in HepG2 cells through determination of cellular viability, MDA, GSH, the activity of anti-oxidant enzymes including GPx, CAT, and SOD, as well as the gene expression of NF-κB, TNF-α, IL-6, and DPP-4. This work provides substantial insight into the therapeutic use of AP and its rich sources to avoid diabetes-related oxidative stress complications.

## Materials and Methods

***Chemicals and reagents***

Alpha-pinene (≥98.0%) and D-glucose were obtained from Sigma Aldrich Chemicals Co. (USA); Roswell Park Memorial Institute Medium (RPMI), fetal bovine serum (FBS), penicillin, and streptomycin were provided by Gibco-BRL (Paisley, UK). Trypsin was from BDH, England. Bovine serum albumin (BSA), ethylenediaminetetraacetic acid (EDTA), 3-(4,5-Dimethylthiazol- 2-yl)-2,5-diphenyltetrazolium bromide (MTT), and dimethyl sulfoxide (DMSO) were purchased from Sigma Aldrich Chemical Co. (UK). Asanzol (Trizol) reagent and commercial kits for the biochemical assessment of MDA, GSH, CAT, GPX, and SOD were taken from Aryagen Sobhan Azma Novin (Asan) Co. (Khorramabad, Iran). The complementary DNA (cDNA) synthesis kit and SYBR Green qPCR Master Mix 2x were from Yekta Tajhiz Azma (YTA) Co. (Iran). Also, the specific forward and reverse primers were obtained from Sinaclon Co. (Iran). The rest of the reagents and chemical compositions are identified in the paper.

***Cell culture and treatment***

The human HepG2 cell line was provided by the National Cell Bank of Iran (NCBI, Pasteur Institute, Tehran). Cells were subsequently grown in RPMI 1640 medium with 10% heat-inactivated FBS, 100 IU/ml penicillin, and 100 μg/ml streptomycin in a humid incubator containing 5% CO2 at 37 °C until they grew to a confluence of at least 80%. The cell monolayer was detached using trypsin-EDTA to obtain single-cell suspensions, and viable cells were counted using a hemocytometer and diluted with medium to give a final density of 1x10^6^ cells for any experiments. The medium was changed every 3–4 days. All experiments were carried out with cells that were 70% confluent (21). The cells were treated with non or HG concentration (50 mM) in the absence or presence of AP at safe doses (8, 16, and 32 μg/ml) for 48 hr according to our MTT assay findings. AP was dissolved in DMSO, and then serial dilutions in RPMI were prepared. The final DMSO concentration was < 1%, which did not affect cell growth.

***MTT assay for cell viability***

HepG2 cells were seeded in 96-well plates at a density of 2×10^4^ cells/well and grown with a maintenance medium for 24 hours to determine cytotoxicity. Following that, they were exposed to various glucose concentrations (0, 20, 30, 40, 50, and 60 mM) for 24, 48, and 72 hr or different AP concentrations (0, 0.5, 1, 2, 4, 8, 16, 32, and 64 μg/ml) for 48 hr. To evaluate cell viability, the colorimetric MTT test was used. Accordingly, 2 mg/mL MTT solution was added and incubated at 37 °C for 4 hr. The MTT and medium were removed from each well without disturbing the cells, and the generated formazan crystals were dissolved in 100 µl DMSO. Finally, the absorbance was measured using an ELISA reader (Bio-Rad, USA) at 570/650 nm wavelength. The cell viability was determined as the ratio of absorbance of treated cells to that of untreated cells that served as a control.

***Cell lysate preparation***

The treated HepG2 cells were cleaned and collected with PBS. Subsequently, the cell suspension was centrifuged for 10 min and kept at –20 °C until use. Cells were lysed using protein extraction buffer to perform biochemical evaluations, then sonicated for 2 min on ice. After centrifugation of the mixture for 10 min, the supernatant was analyzed for biochemical variables.

***MDA determination***

MDA levels, as an index of lipid peroxidation, were estimated based on the thiobarbituric acid (TBA) assay according to the Esterbauer and Zollern method with minor change (24). At first, 100 µl of the cell media sample was added to the tube containing 2.5 ml trichloroacetic acid (TCA)-TBA-HCl reagent and mixed thoroughly. Then, it was incubated in boiling water for 30 min and centrifuged at 1000 RPM for 15 min. The OD of samples was read at 593 nm using a spectrophotometer. The test was carried out three times for each sample. The MDA levels were reported as nmol/mg protein.

***Reduced GSH content determination***

Glutathione content was quantified according to the Rahman *et al*. method (25) with slight alterations. Accordingly, 25 µl samples were added to an ELISA microplate. Then, 30 µl of 0.1M DTNB and 140 µl of 0.2M tris-EDTA (pH 8) were added. Finally, the absorbance of the wells was read using an ELISA reader at 412 nm wavelength. The test was run three times for each sample, and the amounts of GSH were reported as µmol/mg protein.

***Measurement of GPx activity***

The GPx activity was estimated by the Flohé and Günzler method (26). First, 200 µl of 0.4 M tris-HCl, pH 7, 100 µl of 1 mM NaN3, and 200 µl of samples were mixed in a tube. Then, 200 µl of 2 mM glutathione and 100 µl of 0.2 mM H2O2 were added to the mixture and incubated at 37 °C for 10 min. After that, 0.4 ml 10% TCA was added and centrifuged at 2000 rpm for 3 min. Then, 25 l of supernatant was added to an ELISA microplate, followed by 140 l of 0.2M tris-EDTA (pH 8) and 30 l of DTNB, respectively. After 30 min of incubation at room temperature, the absorbance of the samples was measured at 420 nm vs a blank using an ELISA reader in triplicate. The activity levels of GPx are expressed as U/mg protein.

***Measurement of CAT activity***

The method of catalase activity was followed by the Aebi method (27), as mentioned previously. At first, 50 µl of the sample and 1ml of potassium phosphate (50 mM, pH 8) were mixed in a tube. Then 50 ml H2O2 was added to the tubes, and absorbance of the samples was recorded at 240 nm using a spectrophotometer vs a blank for 0, 30, and 60 sec. Eventually, the CAT activity was expressed as U/mg protein.

***Measurement of SOD activity***

The activity of the SOD enzyme was determined using the Sun *et al.* method (28) as previously described. At first, the spectrophotometer was set with a tris-EDTA buffer (pH 2.8); 1000 µl of the tris-EDTA buffer, 50 µl of ddH2O, and 1000 µl of pyrogallol 0.2M were added to the cuvette and its absorbance was read at time 0 and 1 min. The absorption difference at 0 and 1 min was considered as a control. For samples, 50 ml of serum was added to the cuvette instead of distilled water, and its absorbance was read at 0 and 1 min. The following formula was used to determine the percentage of inhibition of pyrogallol autoxidation: (Absorbance of the sample at 0 time - absorbance of the sample at 1 min)/(Absorbance of the control at 0 time - absorbance of the control at 1 min). Then, the amount of enzyme activity was obtained in terms of U/ml by the mentioned formula: The percentage of inhibition of pyrogallol autoxidation/50%.

***Determination of total protein***

The Bradford method was employed to assess the protein content (mg/ml), and bovine serum albumin (BSA) was used as a reference (29).

***Real-time PCR***

Total RNAs were separated from HepG2 cells using Asanzol (Trizol) reagent (Asan, Khorramabad, Iran), and cDNA was prepared using a cDNA Synthesis Kit (Cat No. YT4500, YKA, Tehran, Iran) according to the manufacturer’s guidelines. The quantitative real-time polymerase chain reaction (qRT-PCR) was used to evaluate gene expression using SYBR Green qPCR Master Mix 2x (Cat No. YT2551, YTA, Tehran, Iran) and the defined forward and reverse primers (Sinaclon, Tehran, Iran). The primer sequences (Table 1) was determined using data from the gene bank of the National Centre for Biotechnology Information (NCBI) public database. The data were normalized to the housekeeping gene GAPDH, and fold-change in expression (expression levels) was estimated using the 2^-ΔΔCt^ method. 

***Statistical analysis***

Statistical analyses and illustration were done using SPSS 22.0 (SPSS, Chicago, IL, USA) and GraphPad Prism version 7.0 (GraphPad Software Inc). Comparisons between groups were made using one-way analysis of variance (ANOVA), followed by the Tukey test as a post hoc analysis. All results were presented as the mean ± standard deviations (SD) of three separate tests. Statistical significance was defined as values of *P*<0.05.

## Results

***Characterization of dose-*** ***and time-dependent toxicity of glucose in HepG2 cells***

**Figure 2:**
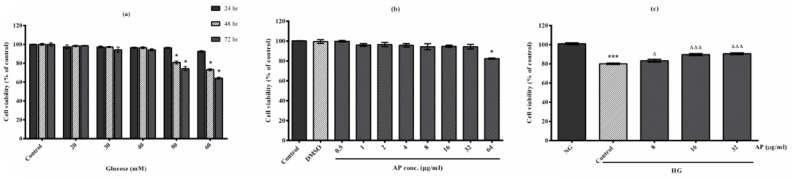
Effects of high glucose (HG) conditions and alpha-pinene (AP) on cytotoxicity and cytoprotection in HepG2 cells

At first, the cytotoxicity of various glucose concentrations on cellular viability was evaluated using an MTT assay. As demonstrated in Figure 2a, exposure of HepG2 cells with HG concentrations induced cell death in a dose- and time-dependent manner. A significant decrease in viability was observed at 48 and 72 hr when the cells were incubated with 50 or 60 mM glucose. Since similar toxicity was observed at both concentrations at both time points, we used the lower concentration, i.e., 50 mM glucose, to treat HepG2 cells and a period of 48 hr for subsequent experiments. 

***Cytotoxicity and cytoprotective effects of AP on the viability of HepG2 cells***

To investigate the safe dose of AP, we cultured the cells with various concentrations of AP (0.5–64 μg/ml) for 48 hr. As shown in Figure 2b, AP did not induce significant toxicity at concentrations up to 32 g/ml in HepG2 cells. Given this result, 8, 16, and 32 μg/ml of AP were chosen as non-cytotec doses for subsequent experiments. Our findings in Figure 2c demonstrated that induction of glucose (50 mM) for 48 hr caused a reduction in the viability of the HepG2 cells (20.63%) compared with the untreated cells. In addition, AP at 8, 16, and 32 µg/ml doses significantly increased cell viability for 48 hr in HG cells compared with control ones (4.04%, *P*<0.05; 11.94%, *P*<0.001; 13.15%, *P*<0.001, respectively). A maximal protective effect was observed at 32 μg/ml of CT. 

***Effect of glucose and AP on MDA levels in HepG2 cells***

**Figure 3:**
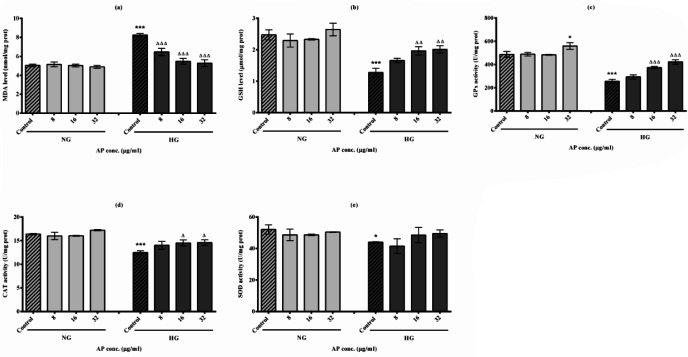
Effects of high glucose (HG) conditions and alpha-pinene (AP) on oxidative stress markers in HepG2 cells

As seen in Figure 3a, a substantial rise in MDA production was observed in HG control (HG-C) cells compared with NG control (NG-C) cells (*P*<0.001). There was no significant difference in MDA levels following the exposure of AP in the NG group. On the other hand, AP treatment in a dose-dependent manner markedly decreased MDA levels in HG-treated cells compared with control ones (*P*<0.001).

***Effect of glucose and AP on GSH levels in HepG2 cells***

Our findings in Figure 3b demonstrated that GSH levels significantly reduced cells with HG conditions compared with NG-C cells (*P*<0.001). Although GSH levels were not considerably affected after the treatment with AP in NG cells, we have seen that AP at the higher doses (16 and 32 µg/ml) could significantly increase the levels of GSH in the HepG2 cells under HG conditions compared with HG-C group (*P*<0.01). 

***Effect of glucose and AP on GPx enzyme activity in HepG2 cells***

As we see in Figure 3c, the level of GPx enzyme activity decreased significantly in HepG2 cells after HG induction compared with NG-C cells (*P*<0.001). In contrast, NG cells treated with higher concentrations of AP (32 µg/ml) exhibited increased activity of GPx activity compared with controls (*P*<0.05). Similarly, the higher doses of AP (16 and 32 µg/ml) also enhanced GPx activity in HG cells compared with the HG-C group (*P*<0.001).

***Effect of glucose and AP on CAT enzyme activity in HepG2 cells***

The results of our study in Figure 3d show that the induction of HG conditions significantly reduces the level of CAT enzyme activity compared to the control group (*P*<0.001). Although AP could not considerably change the CAT activity in the NG group, it was able to significantly improve the reduced activity level of this enzyme at the higher doses (16 and 32 µg/ml) in HepG2 cells under HG conditions compared to the control ones (*P*<0.05).

***Effect of glucose and AP on SOD enzyme activity in HepG2 cells***

As demonstrated in Figure 3e, induction of HG conditions significantly reduced the activity level of SOD in comparison to the NG-C group (*P*<0.001). However, no significant difference was seen in enzyme activity after treatment with AP in the NG or HG groups compared with their control ones (*P*>0.05).

***Effect of glucose and AP on TNF-α and IL-6 gene expression in HepG2 cells***

**Figure 4:**
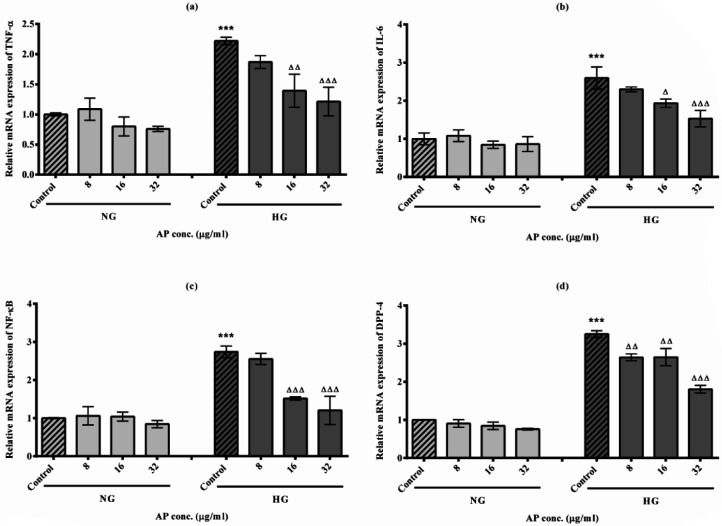
**. **Effects of high glucose (HG) conditions and alpha-pinene (AP) on gene expression inflammatory biomarkers in HepG2 cells

The outcomes are displayed in Figure 4a, b. The gene expression levels of TNF-α and IL-6 were considerably increased in the HG-C group compared with the NG-C group (2.2 fold, *P*<0.001 and 2.5 fold, *P*<0.001, respectively). No significant alterations in gene expression were observed in the NG group following the treatment with AP. Furthermore, AP treatment at the higher doses (16 and 32 µg/ml) in the HG group led to a substantial decrease in gene expression of TNF-α (37.38%, *P*<0.01 and 45.49%, *P*<0.001, respectively) as well as IL-6 (25.48%, *P*<0.05 and 40.92%, *P*<0.001, respectively) compared with HG-C group. 

. 

***Effect of glucose and AP on NF-*** ***κB*** ***gene expression in HepG2 cells***

Findings in Figure 4c demonstrated that the gene expression of NF-κB in the HG-C group increased significantly compared to the NG-C group (2.7 fold, *P*<0.001). Although the gene expression of NF-B did not alter significantly after treatment with AP in the NG group, it can be seen that AP at the higher concentrations (16 and 32 µg/ml) significantly reduced the NF-κB gene expression in the group under HG conditions in comparison to the control group (44.68%, *P*<0.001 and 56.04%, *P*<0.001, respectively).

***Effect of glucose and AP on DPP-4 gene expression in HepG2 cells***

As seen in Figure 4d, HG conditions induced markedly the gene expression of DPP-4 in HepG2 cells compared to the NG-C group (*P*<0.001). We have not seen any considerable change in the expression level of this gene among the NG group treated with AP. But AP treatment with all doses (8, 16, and 32 µg/ml) in HG cells led to a significant reduction in DPP-4 gene expression compared to the HG-C group (18.76%, *P*<0.01; 18.76%, *P*<0.01; 44.61%, *P*<0.001, respectively).

**Table 1:** Primer sequences for Homo sapiens used in qRT-PCR

**Gene**	**Forward sequence**	**Reverse sequence**	**Product size (bp)**	**Accession No**
**TNF-α**	5′-CCCAGGGACCTCTCTCTAATC-3′	5′-ATGGGCTACAGGCTTGTCACT-3′	84	NM_000594.4
**IL-6**	5′-TGAACTCCTTCTCCACAAGCG-3′	5′-TCTGAAGAGGTGAGTGGCTGTC-3′	151	NM_000600.5
**NF-kB**	5′-AACAGAGAGGATTTCGTTTCCG-3′	5′-TTTGACCTGAGGGTAAGACTTCT-3′	104	NM_001382627.1
**DPP-4**	5′-AAGATGGAACTGCTTAGTGG-3′	5′-TAGAGCTTCTATCCCGATGAC-3′	226	NM_001379604.1
**GAPDH**	5′-CGACCACTTTGTCAAGCTCA-3′	5′-AGGGGTCTACATGGCAACTG-3′	228	NM_001357943.2

(a) Cytotoxicity of different concentrations of glucose in HepG2 cells for 24, 48, and 72 hr. (b) Cytotoxicity of various concentrations of AP in HepG2 cells for 48 hr. (c) Viability of HepG2 cells following AP protection and HG damage. All data were presented as mean ± SD of three independent tests. The significant difference between treatment and control groups was determined using one-way ANOVA, followed by Tukey’s test. * *P*<0.05; **P<0.01; *** *P*<0.001, treated vs untreated cells in NG condition; and control in HG vs control in NG. ^Δ^ *P*<0.05; ^ΔΔ^ *P*<0.01; ^ΔΔΔ^ *P*<0.001, treated vs untreated cells in HG condition

(a) Malondialdehyde (MDA) level. (b) Glutathione (GSH) level. (c) Glutathione peroxidase (GPx) Activity. (d) Catalase (CAT) activity. (e) Superoxide dismutase (SOD) activity

All data were presented as mean ± SD of three independent tests. The significant difference between treatment and control groups was determined using one-way ANOVA, followed by Tukey’s test. * *P*<0.05; **P<0.01; *** *P*<0.001, treated vs untreated cells in NG conditions; and control in HG vs control in NG. ^Δ^*P*<0.05; ^ΔΔ^*P*<0.01; ^ΔΔΔ^*P*<0.001, treated vs untreated cells in HG conditions.

(a) Tumor necrosis factor-alpha (TNF-α). (b) Interleukin-6 (IL-6). (c) Nuclear factor-κβ (NF-κB). (d) Dipeptidyl peptidase-4 (DPP-4). All data were presented as mean ± SD of three independent tests

The significant difference between treatment and control groups was determined using one-way ANOVA, followed by Tukey’s test. * * P*<0.05; ** P<0.01; *** * P*<0.001, treated vs untreated cells in NG conditions; and control in HG vs control in NG. ^Δ^* P*<0.05; ^ΔΔ^ *P*<0.01; ^ΔΔΔ^ *P*<0.001, treated vs untreated cells in HG conditions.

## Discussion

Given the growing number of diabetic patients globally, developing appropriate treatment techniques to reduce complications in diabetes mellitus has become a major priority (15). Natural chemicals are still valuable for developing medications (12, 13). AP, a well-known secondary metabolite (monoterpenes) generated from plants, has received great attention for its potential use in medicine, industry, and commerce (17, 18). Some studies illustrated the medical potential of AP due to its pharmacological and biological activities (30-32). However, as far as we know, there is no report evaluating the impact of AP on HG-induced oxidative stress. To make an *in vitro* model of glucose toxicity in hepatocytes, we employed the HepG2 cell line and HG concentration. According to several publications, hepG2 cells are a well-characterized cell line for *in vitro* models of hyperglycemia research (7, 19-23). Considering MTT assay findings, HG concentration at different dosages and periods has varying impacts on cellular viability patterns. Cells exposed to 50 mM glucose for 48 hr displayed considerable damage, indicating that this circumstance was appropriate for setting an HG damage model with cell viability around 80%, which was employed for subsequent tests. Several studies have utilized a high glucose concentration (50 mM) as an *in vitro* model to investigate toxicity caused by hyperglycemia that simulated the *in vivo* state of diabetic ketoacidosis in acute or untreated diabetes (19, 20, 33). In this study, we also administrated the non-toxic concentrations of AP (8, 16, and 32 μg/ml) to evaluate its cytoprotective effects in HepG2 cells. Previous investigations have indicated that HG concentrations can induce hepatic cell injury, leading to increased cell apoptosis in diabetes (19-21). Our findings demonstrated that AP could protect HepG2 cells against HG-induced death in a dose-dependent manner. Similarly, Bae G *et al*. noted that *in vitro*, AP reduced cerulein-induced death in pancreatic acinar cells exposed to isolated cerulein (30). In another report by Porres-Martínez *et al*., the pre-administration of AP (79.70 mM) prevented U373-MG cells from H2O2-induced oxidative stress by suppressing cell death, ROS production, and lipid peroxidation (34). It has also been shown that AP administration inhibited UVA-stimulated cytotoxicity by reducing HaCat cell death caused by UVA exposure (31). So, these results point out that AP can protect cells from oxidative stress-induced damage and death, which may be caused by interfering with oxidative stress and cell apoptosis pathways (34, 35).

Our findings also revealed that HG-stimulated cell oxidative damage promoted MDA production in HepG2 cells. This observation confirmed the result of the previous studies that 50 mM glucose treatment enhanced lipid peroxidation through accumulation of free radicals (19, 21, 22). Lipid peroxidation is one of the main intermediary processes in oxidative stress-induced cellular damage (4). Therefore, modulation of MDA production as a lipid peroxidation marker is essential in preventing HG-induced oxidative stress. Based on the attained results, administering AP in all doses notably reduced the concentration of MDA in HepG2 cells exposed to HG. This finding is consistent with previous research showing that AP can considerably inhibit lipid peroxidation at 10 and 25 mM doses in H2O2-treated cells (34). Likewise, it has also indicated that AP treatment before UVA exposure inhibited lipid peroxidation markedly in HaCaT cells (31). Additionally, co-treatment with AP suppressed the aspirin-induced lipid peroxidation in the IEC-6 cells (36). Other monoterpenes, such as camphene, geranyl acetate, and p-cymene, have already been shown to have anti-lipid peroxidative properties (37). As a result, we inferred that reduced HG-induced lipid peroxidation may be ascribed to AP’s free radical scavenging and anti-oxidant capacity. So, co-treatment with AP inhibited the development of lipid peroxidation in HG conditions.

A vital part of the defense against liver injury with HG levels is the cellular anti-oxidant system. As expected, we have seen a depletion in the GSH concentration and GPx, CAT, and SOD activities after treatment with HG concentrations in human hepatocytes, consistent with the previous reports (20, 23, 33). The inherent defense mechanism in HepG2 cells is activated by HG exposure, and anti-oxidant enzymes work to remove more exogenous glucose (38). The decreased anti-oxidant enzyme activity in HepG2 cells treated with HG might be attributed to oxidative stress-induced cell death. Moreover, GSH reduction represents intracellular oxidation, while a sufficient GSH content is necessary to prepare the cell against an oxidative shock (33, 36, 39). Conversely, we found that AP administration restored the anti-oxidant system, GSH, GPx, CAT, and SOD, in HepG2 cells against HG-induced oxidative stress, although the increase in SOD activity was insignificant. This finding supports earlier research by Karthikeyan *et al*., who showed that AP treatment significantly improves the SOD, CAT, GPx activity, and GSH levels in mouse skin exposed to UVA (39). similarly, the co-exposure of AP (0.5 mg/ml) on aspirin-induced toxicity in IEC-6 cells showed a considerable rise in the GSH level (36). Recently, citral, a monoterpene, has been identified as enhancing anti-oxidant enzyme activity preserving hepatocytes against HG concentrations (33). Moreover, the previous findings reported that AP has substantial oxygen radical scavenging capacity and modulates oxidative stress by suppressing ROS formation (31, 34, 36). So, it can be concluded that AP protects the anti-oxidant defense system in human hepatocytes.

One of the most apparent and rapid reactions of cells exposed to HG conditions is an inflammatory response accompanied by the stimulation of several signaling pathways. Activation of NF-κB is the hallmark of host cell response to HG-induced oxidative stress, which releases pro-inflammatory cytokines, including IL-6 and TNF-α, resulting in inflammation (7). According to our data, the NF-κB, TNF-α, and IL-6 gene expression was noticeably raised by HG conditions in HepG2 cells. Meanwhile, co-administration of AP (16 and 32 μg/ml) significantly reduced the up-regulated NF-κB activity and pro-inflammatory cytokines in hepatocytes. Similarly, Karthikeyan *et al*. demonstrated that AP prevents the gene expression of UVA-stimulated inflammatory mediators such as NF-κB, TNF-α, and IL-6 in HaCaT cells (31). Researchers demonstrated that AP could suppress the release of IL-6 and TNF-α by suppressing NF-κB in a dose-dependent manner in LPS-stimulated macrophages (40). There is also existing evidence that indicates the inhibitory effect of AP on the translocation of NF-κB/p65 protein induced by LPS in THP-1 cells (32). Furthermore, AP administration reduced the production of pancreatic TNF-α and IL-6 during cerulein-induced acute pancreatitis (30). Monoterpenes derived from natural medicinal plants have been identified as promising anti-inflammatory medicines that can alter key signaling molecules implicated in inflammation. Moreover, J.S.S. Quintans *et al*., in a review study, evidenced that NF-B signaling is one of the most crucial mechanisms for the anti-inflammatory effects of monoterpenes (41). As a result, the potential anti-inflammatory impact of AP in hepatocytes caused by HG may be strongly linked to the suppression of NF-κB. Furthermore, the preventive effect of AP in HG-induced lipid peroxidation has been associated with a reduction in the production of lipid mediators, including prostaglandins, which play a crucial role in the inflammatory response (31). Hence, it can be inferred that AP can impede the inflammatory response generated by HG via blocking NF-κB and exhibiting anti-oxidant features. These findings indicate that AP could be a novel therapeutic agent for alleviating inflammatory ailments.

DPP-4 is widely recognized for its critical function in controlling glycemia through incretin peptide metabolism (10). It also has influences on possible pathophysiological roles in metabolic and inflammatory disorders. DPP-4 is found in different kinds of cells and tissues, including the kidney and liver (10, 42). Our experiment showed that the expression of DPP4 was augmented by HG conditions and conversely modulated by treatment with AP in a dose-dependent manner. The previous works similarly found an increscent in DPP-4 gene expression in HepG2 cells exposed to HG concentration (9, 10). Molecular docking studies propose using herbal DPP4-inhibitors as a diabetic treatment target, with terpenoid components being the key active ingredient (43, 44). LC-MS analysis and *in silico* docking studies have indicated the inhibitory effect of the ethanolic extract of Urena lobata leaves, which may be attributed to the high content of terpenoids (43). In the same way, a molecular docking study suggested that pentacyclic terpene compounds in I. obliquus mycelium powders could also prevent DPP-4 activity (44). Various research has demonstrated anti-oxidant effects in natural DPP-4 inhibitors derived from plants (45, 46). So, it may be inferred that AP exhibits both DPP-4 inhibitory actions and anti-oxidant capabilities. However, the relevant mechanisms and variables involved in the function of the DPP-4 enzyme are very complicated and need more clarification.

## Conclusion

We provided evidence that AP exerts protective actions against HG-induced injuries to human hepatocytes in a dose-dependent manner by reducing oxidative stress and inflammatory responses, most likely due to its anti-oxidant features. Future studies are warranted to explore the impact of AP on diabetes consequences and to find the other mechanisms of action in ameliorating oxidative stress and inflammation in cells under HG conditions. 

## Authors’ Contributions

R C, M A, and H A contributed to the conception and design of the study; R C and M A performed experiments and collected data; R C, A A, and H A discussed the results and strategy; H A supervised, directed, and managed the study; A A and F E contributed to data analysis. R C and H A wrote the manuscript. The final version of the manuscript was reviewed and approved by all authors.

## Conflicts of Interest

The authors state no conflicts of interest.
